# Regulatory framework for genetically modified organisms in the Kingdom of Eswatini

**DOI:** 10.1080/21645698.2024.2375664

**Published:** 2024-07-04

**Authors:** Bongani Z. Nkhabindze, Cebisile N. Magagula, Diana Earnshaw, Calsile F. Mhlanga, Sipho N. Matsebula, Isaac G. Dladla

**Affiliations:** a Department of Crop Production, Faculty of Agriculture, University of Eswatini, Luyengo, Eswatini; b Department of Biological Sciences, Faculty of Science and Engineering, University of Eswatini, Kwaluseni, Eswatini; c Department of Natural Resource Management, Eswatini Environment Authority (EEA), Mbabane, Eswatini

**Keywords:** Eswatini, biosafety, living modified organisms, genetically modified organisms

## Abstract

The Kingdom of Eswatini is a Party to the Convention on Biological Diversity and to the Cartagena Protocol on Biosafety. As Party, Eswatini has domesticated these agreements by passing the Biosafety Act, of 2012 to provide for the safe handling, transfer, and use of living modified organisms (LMOs) in the country. The Act regulates living modified organisms to be used for confined field trials, commercial release, import, export, and transit, and for food, feed, and processing. Guidance is provided for prospective applicants before any application is made to the Competent Authority. This framework also provides for the regulation of emerging technologies such as synthetic biology and genome editing. The regulatory framework for living modified organisms aims to provide an enabling environment for the precautionary use of modern biotechnology and its products in the country in order to safeguard biological diversity and human health.

## 1. Introduction

Crop improvement began at the time when human beings selected superior plants to domesticate and grow. Crop selection was the main tool to sustain their livelihoods and to ensure food security in their societies.^1^ Using phenotypic features like high-yielding, bigger-sized products, and attractive color, the breeders used their experience to ensure genetic improvement.^2,3^ A classic example of crop selection is how *Zea mays* evolved from teosinte to what we know to be maize today.

There then followed the use of hybridization techniques where humans began breeding crops, mainly within the species. These hybridization techniques continuously improved crops by breeding for specific superior characteristics like drought tolerance, pest resistance, high yielding (heterosis), disease resistance, and so on.^4^ These characteristics could be sourced from other varieties or wild relatives of those crops, or even from spontaneous mutants. The practices of selection, hybridization, and mutation are collectively referred to as conventional plant breeding techniques. These crop improvement methods have been ongoing without being monitored by any regulatory framework or even criticism.^1^

Crop improvement techniques then evolved to the manipulation of the nucleic acids in the organisms by directly moving the genes that influence that particular characteristic and introgressing them into the target crop in order to improve it for that particular characteristic. Existing variability within a species may not be sufficient for crop improvement, and many traits may be imported from distantly related or unrelated organisms.^5^ This technique, referred to as transgenic hybridization or modern biotechnology, capitalizes on the fact that DNA is universal in all organisms, as they all have the same bases A, T, C, and G. Modern biotechnology is referred to “as the application of in-vitro nucleic acid techniques, including recombinant deoxyribonucleic acid (rDNA) and direct injection of nucleic acid into cells or organelles, or the fusion of cells beyond their taxonomic families,”^6^ resulting in living modified organisms (LMOs).

The advances in modern biotechnology have enabled the improvement of crops through the development of resistance to both biological and non-biological challenges, as well as developing agricultural resilience to the effects of climate change.^7^ This in-turn enhances agricultural production globally. The global adoption of GM crops has reached more that 191 million hectares of commercially grown GMO crops, while Africa contributes more than 3 million hectares, and Eswatini, having commercialized GM cotton, grows more than 300 hectares.^8,9^

Due to the novel nature of LMOs, Article 8(g) of the Convention on Biological Diversity calls for Parties to “regulate and control living modified organisms, as products of modern biotechnology the products, which may cause adverse environmental impacts that could affect the conservation of and sustainable use of biological diversity, taking into account the risks to human health.”^10,11^ The extension of this article gives the legal basis for the establishment of the Cartagena Protocol on Biosafety, as a supplementary protocol to the convention.^6^ The use of modern biotechnology is being regulated globally, and as such, like with any other new technology, its safety concerns, and associated risks are assessed before they are deployed^12^ and the aim of this paper is to present the regulatory framework for such products in Eswatini.

## 2. The Cartagena Protocol on Biosafety

The Cartagena Protocol on Biosafety (CPB) is a multilateral agreement that seeks to ensure adequate safety in the use of LMOs to protect biological diversity and human health, with an emphasis on the transboundary movement.^6^ The CPB uses the precautionary approach in the regulation of the transboundary movement of LMOs. The Kingdom of Eswatini is Party to both the Convention on Biological Diversity, since 1995, and the Cartagena Protocol on Biosafety, since 2006. Parties to the CPB are always encouraged to customize and domesticate the Protocol into their local legislation which they will use to regulate LMOs.^6^

## 3. The Biosafety Act of 2012

Article 2 of the Cartagena Protocol on Biosafety calls for Parties to take necessary and appropriate legal and administrative measures to implement their obligations under the Protocol.^6^ Eswatini, as a Party to both the Convention on Biological Diversity (CBD) and the Cartagena Protocol on Biosafety (CPB), has domesticated these multilateral agreements into the Biosafety Act of 2012. The Biosafety Act defines both living modified organisms (LMOs) and genetically modified organisms (GMOs) under the same definition and also uses the terms interchangeably. The Act aims to ensure safety when using, transferring, or handling LMOs in the country. This Act designates the Eswatini Environment Authority as the Competent Authority (CA) to ensure that it is implemented parallel to the CPB with the objective of protecting biological diversity and human health. This is the guiding document for all activities that include the application process, conducting risk assessments, labeling of LMO products, public awareness, and education, as well as monitoring and enforcement.^13^ The application process outlines the procedures that should be followed whenever there is prospective use of GMOs for any activity which may include intentional introduction to the environment, import, export, and transit, GMO for food, feed, and for processing, as well as for confined field trials (CFTs) within the country. The Biosafety Regulations is the instrument that is used to implement the Act, as they further unpacks the provisions that are in the legislation.

### 3.1. Living modified organisms for contained used

The Cartagena Protocol on Biosafety defines contained use as “any operation, undertaken within a facility, installation or other physical structure, which involves living modified organisms that are controlled by specific measures that effectively limit their contact with, and their impact on, the external environment.” The Biosafety Act calls for every prospecting user to forward an application that will be reviewed by the Competent Authority and a decision should be issued within 60 d.^13^

A newly developed LMO should be evaluated against the closest non-LMO counterpart (near-isogenic line) for its phenotypic qualities and other safety studies.^14^ The event will undergo a number of biosafety studies, then be introgressed into a number of locally adapted non-GM hybrids that will then be evaluated.^15^ These CFTs are conducted in spaces that are not open to the public.^6^ These may be in experimental stations, laboratories, greenhouses, or fenced demonstration plots that do not exceed 5 ha in size.^13^ Conducting the CFTs is a major part of the research and development of LMO products which should be done to ensure the safety of the product before they are deployed for commercial release. These trials are conducted in consideration of the sovereign requirements of the particular country.^2^ The country’s Policy emphasizes the undertaking of one or two seasons to conduct experimental research or demonstrations of the LMOs before they can be introduced into the environment. The local legislation also provides for the transportability of data, especially, to forego the requirement to re-invent the wheel by duplicating studies that have been conducted, especially, in near-similar environments.^13^

### 3.2. Relevant seed laws

Seed laws mainly aim to protect the local farmers from buying poorly performing seed and the seed legislations ensure that the seed is registered and the provided information is true and meets the required international standards.^16^ These laws focus mainly on seed registration and certification to ensure that the varieties conform to being identifiable (distinct), stable, and uniform, also known as the DUS standards.^17,18^

The registration of LMO seeds in several countries has proven to be complex, and this has brought up the importance of extensive stewardship systems which are helpful in tracing the use of LMO varieties, seeds, grain, as well as processed products.^18^ In the Kingdom of Eswatini, the Biosafety Act of 2012 and the Seed and Plant Varieties’ Act of 2000, whose custodian is the Seed Quality Control Services under the Ministry of Agriculture, are both used to regulate seed, although LMO seed is regulated by the Biosafety Act, 2012.^13^

### Intentional introduction to the environment

After conducting the CFTs, the next step is the commercial release of the LMO product. The introduction of the LMO into the environment after all evaluations and trials is also a regulated component under biosafety frameworks.^15^ During the commercial release, the LMO gets to be in direct contact with the public, and there is no limitation on the size of the release.^13^

Before releasing LMOs, the public should be educated on how to manage the GM crop. There should also be frequent monitoring of the areas where the release has been done. Monitoring should be done to evaluate the state of the environment before and after releasing the LMO, thereby ensuring the validity of the risk assessment process.^19^

### 3.4. Import, export, and transit

The exchange of agricultural products, including seeds, goes beyond sovereign territories.^20^ The aim of the Cartagena Protocol on Biosafety puts more emphasis on the regulation of the transboundary movement of living modified organisms. By extension, the Nagoya-Kuala Lumpur Supplementary Protocol on Liability and Redress also seeks to give guidance on the transboundary movement of LMOs.^6^ Eswatini is a net importer of LMOs, which means for every activity that is anticipated locally, there should be an application for the importation of the LMO product. The Act also provides for stakeholders who wish to export the LMOs or transit them through the country with the necessary guidance to undertake those activities.^13^ For every transboundary movement of LMOs, there should be the consideration of the “Advance Informed Agreement,” where the exporting country should await the approval of the importing country before the transboundary movement is undertaken.^6^

### 3.5. LMOs for food, feed, and processing

Food and feed that has been produced from genetically modified plants or animals, either grown locally or outside the country, undergo the regulatory process for LMOs. To classify any food or feed product as an LMO, the low-level presence (LLP) or minimum threshold for GM material is regulated at 1%.^13^ Local industries import LMO products from neighboring countries as raw materials to process them into final products. Mostly, these products include white maize, yellow maize, yellow maize grits, and ground soybeans which are currently not sufficiently produced locally.^13^

## 4. Institutional biosafety committees

All institutions that conduct research on modern biotechnology or with prospective projects that will involve the use of LMOs are guided by the law to establish their Institutional Biosafety Committees.^21^ The main function of the Institutional Biosafety Committee (IBC) is to review project proposals within the institution before any application is made to the CA and also to monitor ongoing research on modern biotechnology and other potentially infectious material.^21,22^ The IBC is responsible to provide institutional guidance with regard to the control of biohazards, biosafety requirements, and the need for ethics approval.^23^ This guidance will ultimately result in a recommendation that will ensure compliance with the country’s statutes and call for an application to be compiled and submitted to the CA. The IBC will also be responsible for the institutional monitoring of the project if it is approved by the CA.

## 5. The national biosafety advisory committee

Section 6 of the Biosafety Act establishes the National Biosafety Advisory Committee (NBAC) which has the responsibility of reviewing the submitted applications, conducting the risk assessment, and issuing recommendations to the CA. This committee is appointed by the Ministry of Tourism and Environmental Affairs to be in office for a term that does not exceed 3 y, which may be renewed.^13^ The NBAC also has the responsibility to advise the CA on matters that relate to modern biotechnology. This committee should consist of members that do not exceed nine, selected from the fields of expertise that include a biodiversity specialist, a crop specialist, a veterinary specialist, an animal scientist, a crop protectionist, an NGO representative, a business sector representative, a farmer representative, a trade specialist (WTO focal point), a food specialist, a health specialist, and a representative from the Ministry of Information, Communication, and Technology. The Biosafety Registrar is the permanent secretariat of the committee.

## 6. The biosafety registry

The Biosafety Registry is manned by the Registrar, housed in the CA, and is tasked with the responsibility of receiving and screening the application before they are received by the NBAC and implementing the Act.^13^ The Registrar also seats as the Secretary of the NBAC and reports to the CA’s Chief Executive Officer and Board of Directors whenever Biosafety matters are concerned. The Biosafety Registry is also responsible for recording, documenting, and reporting on all the activities that take place during the risk assessment process.

## 7. The risk assessment process

This is a structured process that is done in a scientifically sound manner, transparently as well as on a case-by-case basis to assess the safety of LMOs in order to understand their safety for humans, animals, and the environment.^24,25^ Parties use Annex 3 of the Cartagena Protocol on Biosafety in adopting their guiding principles for conducting risk assessments. The Biosafety Act calls for the CA to ensure that risk assessments are conducted adequately for all applications that have been received, these may be applications for CFTs, introduction to the environment, FFPs, Import, Export, and Transit.^26^ The risk assessment and risk auditing shall be conducted in a scientific manner, taking into consideration the information that has been submitted by the applicant as well as any other information that can be found in databases such as the Biosafety Clearing House (BCH) and the Organization for Economic Co-operation and Development (OECD).^13^ Genetically modified crops are subjected to risk assessment for all prospective uses which may include CFTs, commercial release, FFPs, Import, Export, and Transit.^26^ This is a requirement by the law in order to protect biological diversity.

The risk assessment that is conducted in the Kingdom of Eswatini adopts the guidance that has been provided by the SCBD. This guidance identifies five distinct steps to conduct the process (Table 1), and these are the identification of any novel genotypic and phenotypic characters that may have any effect on the environment and human health, evaluation of the likelihood for that particular effect to occur, evaluation of the consequences should these effects occur, the estimation of the overall risk that is posed by the LMO, and a recommendation on whether the risk is manageable and suggests possible mitigation measures with regard to an identified protection goal.^24^ Upon conclusion of the risk assessment and auditing process, the Committee shall provide the CA with a risk assessment report that gives its opinion to the CA on whether a permit should be given or denied, with reasons.^13^

**Table 1.** The stepwise representation of the risk assessment process

Step 1:	Step 2:	Step 3:	Step 4:	Step 5:
IDENTIFY THE PROTECTION GOAL				
Identify novel genotypic and phenotypic characteristics of the LMO that may have an effect on the protection goal	Evaluate the likelihood of adverse effects to occur, taking to account the level of exposure of the prospective receiving environment	Evaluate the consequences in the case where these adverse effects are realized	Estimate the overall risk that is posed by the LMO, based on the likelihood and the consequences	Identify if the risk is acceptable/manageable or not and recommend mitigation strategies for the risks

Notes: The information that is provided by the applicant should be of acceptable scientific quality and relevance. Source: Secretariat of the Convention on Biological Diversity.^24^

## 8. Socio-economic considerations for decision making

The interaction of living modified organisms with the ethical, social, and economic factors in the country is a call from Article 26 of the Cartagena Protocol to be considered during the risk assessment process, in order to influence decision-making.^6^ One of the predominant criticisms in regulatory science has been that the focus is mainly drawn to human health and biodiversity, forgetting the interactions of technology with society in order to take note of their concerns and knowledge of the technology.^27,28^

**Figure 1.**
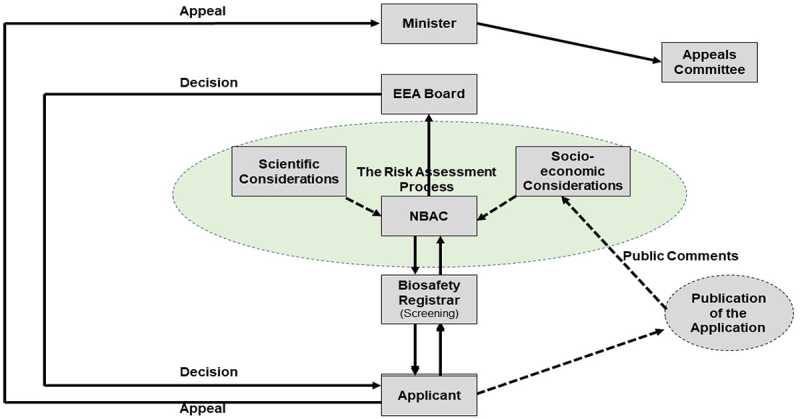
The application process for genetically modified organisms in Eswatini.

Section 4 (3) (h) of the Biosafety Act incorporates socio-economic considerations along with biological diversity and human health as the obligations of the regulator to protect from the adverse effects arising from LMOs. Section 18 calls for the socio-economic considerations to be taken into account during decision-making and communication of decisions. The Act calls for the applicant to publicize their application and then gives the public 28 working days to compile and submit their comments and even objections to the Competent Authority for consideration during the risk assessment process as shown in Figure 1.^13^ Sections 25 and 26 of the Act calls obligate the CA to be transparent in sharing information once it has identified the confidential parts of the application dossier. The Act then calls for the NBAC to review each and every comment, compile them for the applicant to address, and then respond back to the individuals who had submitted them as concerns.^13^

## 9. Handling applications

The Biosafety Act calls for the submission of applications or notifications, at a fee that is prescribed in the Regulations, for all prospective handling, transfer, and use of LMOs in the country and Biosafety Regulations have been formulated in order to facilitate monitoring and enforcement of the Act.

The Biosafety Regulations aims to operationalize the Act by further providing guidance to all prospective GMO users. The applicant has the responsibility to compile the application dossiers as per the schedules in the Biosafety regulations, then submit the application to the Biosafety Registrar, who screens for completeness (Figure 1). If the application is not complete, it is returned back to the Applicant for completion, and if it is complete, the Registrar tables it to the NBAC. The NBAC will then call for the publication of the application in local media, give 28 working days for the public to submit their comments, and then conduct the risk assessment. Upon conducting the risk assessment, the NBAC then compiles its recommended decision to present it to the CA. The CA then issues the final decision to the Applicant which should be within 270 d from the time of application.^13^

## 10. Right of appeal

For any organization to exercise its powers effectively and transparently, it is crucial that applicants be able to question the organization’s decisions since they may significantly affect their reputation, financial viability, and ability to carry out its mission.^29^ An applicant who feels aggrieved by a decision that has been issued by the Competent Authority has the right to submit an appeal directly to the Minister of Tourism and Environmental Affairs within 21 d after the decision has been issued (Figure. 1). To review the appeal, the Minister will appoint a panel of advisors that will include a Socio-economist, a Scientist, and a Legal Expert.^13^

## 11. Monitoring of LMOs

The monitoring of LMOs is a process that is directly linked with the risk assessment process and is imperative in ensuring that the release of the LMO does not have an adverse effect on the environment and human health. Monitoring should be done to evaluate the state of the environment before and after releasing the LMO, thereby ensuring the validity of the risk assessment process.^19,30^ The applicant will submit, along with the application, a management and monitoring plan that will provide the risk assessment process with the proposed mitigation strategies for the perceived risks.^30^

Beyond the monitoring of released events, the presence of genetic modification is constantly monitored to ensure non-contamination of non-LMO consignments as well as to monitor unauthorized LMOs.^31^ The regulatory framework in Eswatini has the allowable low-level presence threshold of GM material to be 1%.^13^

LMO detection and analysis protocols that are effective in the monitoring of LMOs include both nucleic acid-based and protein-based methods.^3^ The designated LMO detection laboratory is situated at the Faculty of Agriculture (Luyengo), University of Eswatini.

The monitoring of LMOs also includes the ecological concerns of gene flow from LMO plants to non-LMO plants that are related to the GM plant.^32^

## 12. Emerging technologies

Globally, there are ongoing discussions on how new breeding technologies, like genome editing (GEd), will be regulated. Genome editing refers to the specific manipulation of the genetic material in living organisms, using site-directed nucleases (SDN), to delete, modify, or insert a DNA sequence typically with the aim of improving that particular crop or farm animal or correcting an existing disorder.^33,34^ The SDNs are used to introduce double stranded breaks (DSBs) at the desired position of the DNA.^35^ Once the DSBs have been introduced, the DNA then tries to repair itself either through a non-homologous end joining (NHEJ) which is prone to errors or a homologous end joining (HEJ) mechanism. The repair process and ligation of the DNA strands result to the deletions and insertions (indels) of nucleotides. The repair mechanism that is used in the HEJ system uses homologous sequences, and this system can be used to incorporate desired genes into the genome with precision.^36^

The NHEJ system results to the modification of the genome without the edition of a foreign gene. The indels in this system are similar to the natural mutation and therefore are categorized as SDN1 and SDN 2 which are regarded as non-LMO in some countries. The HEJ system introduces a foreign gene into the genome, and it is usually categorized as SDN3 and regulated as an LMO.^37,38,^

African countries (Kenya, Nigeria, and Ghana) have adopted GEd and have developed guidelines to use for their assessment. These guidelines have grouped the products of genome editing according to whether a DNA template is used for the modification or if any foreign DNA remains in the genome of the target organism. These group categories are used are SDN1, SDN2, and SDN3.^39,40^

The “SDN1” involves, mostly, the repairing of the genome by editing or by knocking out a gene. This is done mainly by deleting a number of nucleotides at the specifically targeted site. “SDN2” involves the double-stranded break and the use of donor DNA that comes from the same species, with the desired intended improvement. SDN2 mainly introduces base substitutions to the targeted site. “SDN3” involves a double-stranded break and the use of a foreign gene, along with an expression cassette.^40^

Since these GEd processes are developed using different techniques which may or may not require the use of foreign DNA, they may call for the use of different regulatory approaches.^41^

The Biosafety Act of 2012 calls for the regulation of emerging technologies to be in accordance with the definition of LMOs that are provided in the legislation. The legislation also provides for the development of guidelines whenever the need arises.^13^

## 13. Relevance of the regulatory framework

Globally, the world is faced with food security challenges, particularly in sub-Saharan Africa. There is, therefore, a need to increase food production by at least 50% in order to be able to feed the human population by 2050.^42^ To add onto these challenges, currently, food production is faced with the effects of climate change, seasonal changes (especially rainfall), loss of arable land, loss of freshwater resources, the workforce shift from agricultural production, land degradation, urbanization, and the scarcity and price hikes of agricultural inputs.^43^ These pose a threat to conventional agricultural methods of food production, emphasizing the need for innovative methods to ensure productivity.

Modern biotechnology has been one of the innovative solutions that have been availed for adoption to increase agricultural production amid these challenges. The stride toward food security can be achieved by developing and deploying genotypes with higher yield potential, tolerance to environmental stresses to enable cultivation in marginal areas, resistance to insect pests and diseases, improved nutritive value or flavor, drought tolerance, and herbicide tolerance.^9^. As such, the African continent has seen the adoption of insect-resistant and herbicide-tolerant maize in South Africa, Ghana, and Kenya, insect-resistant cotton in Eswatini, Sudan, Kenya, South Africa, and Burkina Faso, and there are still ongoing CFTs of various crops in a number of countries. Farmers from Eswatini are currently lobbying for the commercial release of GM maize, but the maize industry is working on the regulatory requirements. This paper highlights the regulatory framework to facilitate utilization of modern biotechnology in the local environment for food security and economic growth.

Juma and Serageldin^44^ stated that good innovation systems need good governance in order to promote learning and creativity whilst at the same time protecting public interests. The basis of Article 8(g) of the Convention on Biological Diversity falls against this backdrop in its call to regulate and control products of modern biotechnology since they may have adverse effects on the conservation and sustainable use of the environment, as well as human health. The CPB and sovereign regulatory frameworks operationalize the CBD by providing the proper guidance which states need to follow to protect the environment and human health while at the same time reaping benefits from modern biotechnology.

## 14. Conclusions

The regulatory framework of the Eswatini adopts the precautionary principle as outlined in the Cartagena Protocol on Biosafety.^6^ The Biosafety Act calls for the National Biosafety Advisory Committee to conduct risk assessments for all applications that have been received by the CA. When conducting these risk assessments, the committee reviews the scientific and socioeconomic considerations of that particular LMO.^13^ The day-to-day monitoring of activities that involve LMOs remains to be the responsibility of the applicant that will be overseen by the regulator.^30–32,45^
